# Interphase and random nanoscale carbide precipitation in vanadium micro-alloyed steels studied using SANS

**DOI:** 10.1007/s10853-025-10864-z

**Published:** 2025-04-24

**Authors:** Zamran Zahoor Khan, Steven R. Parnell, S. Erik Offerman, Diego Alba Venero, Amir Sabet Ghorabaei, Bart J. Kooi, Niels van Dijk

**Affiliations:** 1 Department of Radiation Science and Technology, Delft University of Technology, Mekelweg 15, 2629 JB Delft, The Netherlands; 2 Department of Material Science and Engineering, Delft University of Technology, Mekelweg 2, 2628 CD Delft, The Netherlands; 3 ISIS Pulsed Neutron and Muon Source, Rutherford Appleton Laboratory, Chilton, Didcot, Oxfordshire OX11 0QX UK; 4 Zernike Institute for Advanced Materials, University of Groningen, Nijenborgh 3, 9747 AG Groningen, The Netherlands

## Abstract

The formation of nanoscale vanadium carbide (VC) precipitates is reported in steels subjected to two different thermal treatments. The thermal treatments lead to either interphase precipitation (IP) or random precipitation (RP). Small-angle neutron scattering measurements coupled with transmission electron microscopy analysis are performed to determine the VC precipitate volume fraction and size distribution. It is seen that the samples exhibiting IP show a higher number density of VC precipitates compared to those undergoing RP. Moreover, a broader size distribution of the precipitate radii is observed in the samples with RP, where lens-shaped nanoscale VC precipitates are found predominantly at grain boundaries (GBs) and sub-grain boundaries (SGBs), with smaller precipitates dispersed within the matrix. It is seen that the addition of carbon and vanadium does not increase the VC precipitate number density when the mechanism of precipitation is IP, whereas an increase in the VC precipitate number density with carbon and vanadium addition is seen in case of RP.

## Introduction

Tackling the problem of climate change demands targeted efforts to address significant contributors to global carbon dioxide (CO_2_) emissions. An important sector in this attempt is the automobile industry, which is recognized as a major source of CO_2_ emissions. The need for innovative and sustainable solutions within this sector is more critical than ever [1]. One promising avenue for mitigating CO_2_ emissions involves using advanced high-strength steels (AHSS) for automobile component manufacturing that offer high strength and good global and local formability allowing for a weight reduction in the vehicle. Conventional multiphase AHSS do not possess such properties, because cracks appear at boundaries separating the hard phase and the soft phase, particularly during stretch-flanging [2]. In contrast, a new generation of AHSS called nanosteels does offer high strength and good global and local formability in a single-phase matrix with nanoscale precipitates [3].

Nanosteels are resource efficient steels that emerged as beneficial alternatives to conventional multiphase AHSS in the production of vehicle components with intricate shapes. These steels possess exceptional properties like high hole expansion capacity, strength and ductility, which can be achieved by the addition of micro-alloying elements such as vanadium (V), titanium (Ti) and molybdenum (Mo) [4]. Nanosteels derive their properties from a single-phase ductile ferritic matrix strengthened by an extremely high number density of nanometer-sized precipitates, making them well-suited for applications in lightweight automotive manufacturing [2, 5, 6].

The nanometer-dimensioned precipitates can form via two mechanisms: interphase precipitation (IP) and random precipitation (RP). Different models have been proposed to explain the mechanism of interphase precipitation [7, 8]. In interphase precipitation, nano-precipitates form during the austenite-to-ferrite phase transformation, along the moving boundary separating the high-temperature austenitic phase from the low-temperature ferritic phase [9]. The moving austenite-ferrite interface is accompanied by precipitate formation in the ferritic matrix. The formed precipitates are generally arranged in a regularly spaced periodic pattern. In random precipitation, the precipitates tend to form at preferred nucleation sites like dislocations and grain boundaries, along with precipitate formation in the matrix [10]. Nucleation at preferred sites like grain corners, edges, dislocations or defects in the material is known as heterogeneous nucleation, whereas if the nucleation process takes place within the matrix it is known as homogeneous nucleation.

This work uses two different thermal processing routes aimed at optimizing the number density of nanosized-VC precipitates in vanadium-alloyed nanosteels in order to enhance their mechanical properties. As is known, steels are often used in conditions where they are subjected to external forces that may lead to plastic deformation. Plastic deformation involves the movement of dislocations in steels. The nanosized-VC precipitates can act as obstacles to these moving dislocations by pinning them, thereby increasing the yield strength of these steels. One of the main goals of this study is to know which of the two precipitation mechanisms results in a higher VC precipitate number density and ultimately higher VC precipitation strengthening. The increase in strength due to the formation of VC precipitates is correlated with precipitate number density, size and the spacing between precipitates. A higher precipitate number density would mean a higher number of pinning sites that inhibits dislocation movement and therefore results in an increase in the yield strength. Precipitation strengthening also depends on the size distribution of these VC precipitates. The moving dislocations interact differently with different sized precipitates. Dislocations tend to bow around the precipitates for bigger precipitates and larger inter-precipitate spacings, whereas they tend to cut through the precipitates for smaller precipitates and shorter inter-precipitate spacings [11]. In this study, we use SANS complemented with TEM to deepen our understanding of the precipitate formation in these nanosteels which opens up new processing routes that enable the optimization of the contribution of the precipitation mechanisms to the strength and formability of nanosteels.

## Experimental

**Figure 1:**
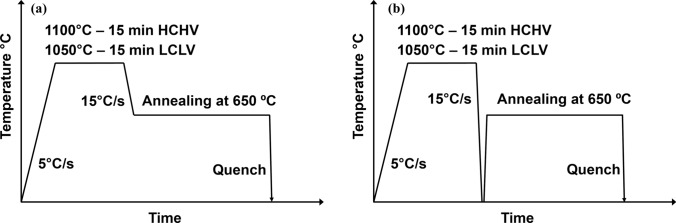
Heat treatments as a function of time applied to study the VC precipitate size distribution for interphase precipitation (left) and random precipitation (right).

Two different nanosteel alloys, one containing relatively low amounts of carbon and vanadium (LCLV) and the other containing relatively high amounts of carbon and vanadium (HCHV), were studied. The alloy compositions are shown in Table 1. The atomic ratio of carbon to vanadium is 1:1 for both alloys. The HCHV alloy contains twice the number of carbon and vanadium atoms as the LCLV alloy. The samples were produced in the form of sheets and were cut mechanically to dimensions of 10 mm × 10 mm × 1 mm. To study the effect of composition and thermal processing on the VC precipitation kinetics, two different types of temperature profiles (Fig. 1) were applied for both alloys, one resulting in VC precipitate formation via the interphase precipitation mechanism and the other resulting in VC precipitate formation via the random precipitation mechanism. A DIL-805 A/D dilatometer (Bähr-Thermoanalysis GmbH) was used for the heat treatment of the samples. First, the samples were heated through inductive heating under a vacuum of $2\times {10}^{-4}$ mbar to the austenitization temperature (1100 °C for the HCHV steel and 1050 °C for the LCLV steel) at a heating rate of 5 °C/s and then held at this temperature for 15 min. Subsequently, for interphase precipitation, the samples were cooled down by helium gas to 650 °C at a rate of 15 °C/s and were annealed for 20 min at this temperature (to produce a mainly ferritic microstructure) before finally quenching to room temperature. For random precipitation, the samples were cooled down from the austenitization temperature to room temperature with the same cooling rate of 15 °C/s (to produce a mainly bainitic/martensitic microstructure) followed by annealing at 650 °C for 20 min before finally quenching to room temperature.

**Table 1:** Chemical compositions (in wt.%) of the studied alloys with balance Fe

Steel		C	Si	Mn	P	S	Cr	Mo	Al	Cu	N	Nb	V
LCLV	wt.% at.%	0.07 0.33	0.010 0.026	1.84 1.86	0.0010 0.0018	0.0016 0.0028	0.010 0.011	< 0.005 < 0.003	0.004 0.008	< 0.005 < 0.004	< 0.001 < 0.004	< 0.0010 < 0.0006	0.29 0.32
HCHV	wt.% at.%	0.14 0.62	0.013 0.026	1.83 1.85	0.001 0.0018	0.0010 0.0017	0.007 0.007	< 0.005 < 0.003	0.008 0.002	< 0.005 < 0.004	< 0.001 < 0.002	< 0.001 < 0.0006	0.57 0.62

TEM studies were performed on carbon extraction replicas (CERs), which were prepared according to a modified approach [12]. A JEOL JEM-2010 TEM was used to investigate the morphology and size distribution of VC nano-precipitates in CERs. Approximately 1500 precipitates, observed from different regions of the corresponding CERs, were considered for statistical analysis.

Room temperature SANS experiments were carried out at the ISIS Neutron and Muon facility in the UK on the Zoom instrument [13] to obtain the differential scattering cross section $(\partial\Sigma /\partial\Omega )$ as a function of the wave-vector transfer $Q$. A neutron beam with a size of 6×6 mm^2^ was used for the SANS experiments. The data reduction in SANS data was done using the Mantid software following conventional procedures [14]. The aim was to study the interphase and random VC precipitation in the LCLV and HCHV steels in the two different heat treatment conditions (Fig. 1). The effect of the precipitation mechanism on the number density, volume fraction and dimensions of VC precipitates was studied, as well as the effect of varying carbon and vanadium contents on the aforementioned quantities.

The SANS data analysis was performed using two methods, fitting using the SASview software package [15] and a Kratky plot analysis [16]. A comparison of the volume fractions of VC obtained by the two methods was made. These values were then compared with results obtained in previous studies [17], from equilibrium calculations performed using the Thermo-Calc Software with the TCFE13 Steels/Fe-alloys database and data obtained from TEM analysis.

## Results

### TEM observations of VC precipitates

**Figure 2:**
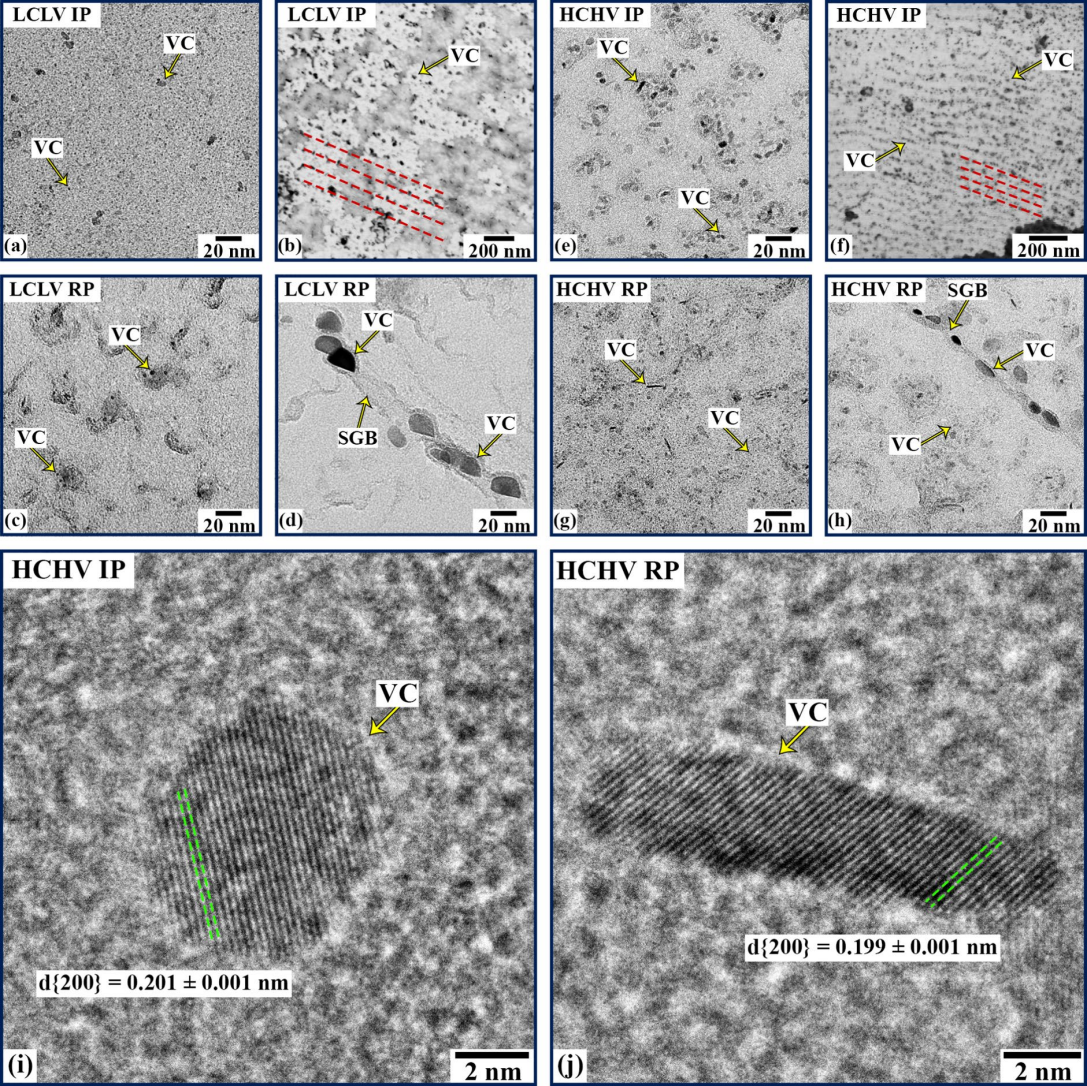
Bright-field TEM images of the CERs for the **a**, **b** LCLV IP (interphase precipitation), **c**, **d** LCLV RP (random precipitation), **e**, **f** HCHV IP and **g**, **h** HCHV RP samples. **i** and **j** HRTEM micrographs of the CERs illustrating lattice images of VC nano-precipitates for the HCHV IP and HCHV RP samples, respectively. SGB: sub-grain boundary of bainite/martensite.

Figure 2 shows bright-field TEM images from the CERs revealing the VC nano-precipitates in the nanosteel samples. The LCLV alloy with interphase precipitation mainly contains spherical/spheroidal VC precipitates (Fig. 2a). Characteristic periodic interphase precipitates along lines (indicated by red dashed lines in Fig. 2b) can also be observed in the CER. For the LCLV alloy with random precipitation, relatively larger VC nano-precipitates are seen in the matrix (Fig. 2c) along with coarser irregular-shaped precipitates decorating sub-grain boundaries of the matrix (Fig. 2d). Similarly, VC precipitates are observed in the matrix as well as at the grain/sub-grain boundaries (GB/SGB) of the HCHV alloy samples as shown in Fig. 2e–h. These precipitates form in three shapes, spherical/spheroidal precipitates located in the matrix, lens-shaped (oblate ellipsoidal) precipitates at GBs and SGBs, and prolate ellipsoidal precipitates that probably nucleated on dislocations. The density of lens-shaped VC precipitates appears to be higher for random precipitation compared to interphase precipitation. In addition, a limited number of prolate ellipsoidal VC precipitates can be seen within the matrix for random precipitation. Typical high-resolution TEM (HRTEM) images from individual VC nano-precipitates in the HCHV alloy samples are provided in Fig. 2i and j, indicating an average VC lattice (NaCl-type) parameter of approximately 0.398 nm. We notice that the VC precipitates formed on the GBs/SGBs in LCLV RP sample are flatter on one side (Fig. 2d), indicating a semi-coherent interface between these precipitates and the grain [18].

### SANS from VC precipitates

SANS is a powerful and non-destructive technique, uniquely suited to unravel the nanoscale VC precipitate size distribution and volume fraction in the studied alloys. In this technique the neutron beam interacts with nanoscale structures (of the order of 1–200 nm), resulting in a 2D pattern of the scattered beam. The resulting 2D scattering pattern contains information regarding the volume fraction, particle size, number density, and composition of nanoscale VC precipitates present in the sample matrix [19]. Moreover, unlike TEM, SANS probes a macroscopic volume, indicative of the bulk sample properties. For smaller VC nano-precipitates, the SANS pattern extends to larger scattering angles. In contrast, grain boundaries, sub-grain boundaries, and dislocations predominantly scatter at smaller angles [20, 21].

To study the size distribution and volume fraction of the nano-scale VC precipitates SANS is used. Using SANS, it becomes possible to extract the precipitate size distribution and volume fraction on a bulk level. The TEM analyzes very small regions of the sample which does not result in good statistics in terms of volume fraction and number density of the precipitates. However, TEM is also used as a complementary technique to know the shape of the precipitates. This is important in analyzing SANS data because in order to accurately extract the precipitate size distribution and volume fraction, a model is fitted to the SANS data. This model is constructed based on the input from the TEM.

The neutron scattering process can be of two types, nuclear and magnetic. Nuclear scattering involves the interaction of neutrons with atomic nuclei and is sensitive to changes in composition within the sample. On the other hand, magnetic scattering involves the interaction of neutrons with the magnetic moments of unpaired electrons within the sample and is thereby sensitive to the density of the magnetic moments. Therefore, the 2D neutron scattering pattern contains contributions from both nuclear and magnetic scattering. To separate the two contributions, an external magnetic field ($\mathbf{B}$) of 1.5 T was applied to the sample during the measurements. This magnetic field aligns the magnetic moments in the ferromagnetic Fe-matrix in the direction of external magnetic field and hence leads to anisotropy in the 2D scattering pattern. The magnetization of the Fe-matrix nearly saturates under the influence of an applied magnetic field of $B$ = 1.5 T. In this case the neutron scattering profile corresponds to:

1 $$\left( {\frac{\partial \Sigma }{{\partial \Omega }}} \right)\left( Q \right) = \left( {\frac{\partial \Sigma }{{\partial \Omega }}} \right)_{{{\text{nuc}}}} \left( Q \right) + \left( {\frac{\partial \Sigma }{{\partial \Omega }}} \right)_{{{\text{mag}}}} \left( Q \right) \sin^{2} \alpha$$

where $\left(\partial\Sigma /\partial\Omega \right)\left(Q\right)$ is the differential scattering cross section [${\text{cm}}^{-1}$], ${\left(\partial\Sigma /\partial\Omega \right)}_{\text{nuc}}\left(Q\right)$ is the nuclear differential scattering cross section, ${\left(\partial\Sigma /\partial\Omega \right)}_{\text{mag}}\left(Q\right)$ is the magnetic differential scattering cross section, $Q$ $=|\mathbf{Q}|=(4\pi /\lambda )\text{sin}\theta$ is the wave-vector transfer [${\text{\AA}}^{ - 1}$], $\theta$ is half the scattering angle and $\alpha$ is the angle between the wave-vector transfer $\mathbf{Q}$ and the external magnetic field $\mathbf{B}$. Next, two sectors of 30 $^\circ$ each, one parallel to $\mathbf{B}$ and the other perpendicular to it are drawn on the 2D pattern. The sector parallel to $\mathbf{B}$ only probes the nuclear scattering, whereas the sector perpendicular to $\mathbf{B}$ probes the sum of the nuclear and the magnetic scattering. It becomes then possible to obtain ${\left(\partial\Sigma /\partial\Omega \right)}_{\text{mag}}\left(Q\right)$ by subtracting the former from the later [19, 22].

Information on the nanoscale VC precipitation mechanism in steels can be obtained by analyzing both the nuclear and magnetic differential scattering cross sections. The following assumptions were made in the data analysis of the SANS signal. The crystal structure of the VC precipitate is assumed to be face-centered cubic (FCC, *Z* = 4) with a lattice parameter *a* = 4.162 Å for the stoichiometric composition (VC) [20]. The crystal structure of the Fe-based matrix is body-centered cubic (BCC, *Z* = 2) with a lattice parameter *a* = 2.867 Å [23]. The nuclear contrast, which is defined as the square of the difference in average scattering length density of the precipitate compared to that of the matrix, is calculated using the following formula:

2 $${\Delta }\rho^{2}_{{{\text{nuc}}}} = (\rho_{p} - \rho_{m} )^{2} = \left[ {\mathop \sum \limits_{{i_{{\text{p}}} }} N_{{0,i_{{\text{p}}} }} b_{{{\text{c}},i_{{\text{p}}} }} - \mathop \sum \limits_{{i_{{\text{m}}} }} N_{{0,i_{{\text{m}}} }} b_{{{\text{c}},i_{{\text{m}}} }} } \right]^{2}$$

where ${i}_{\text{p}}$ and ${i}_{\text{m}}$ refers to the different atomic species present in the precipitate and the matrix, respectively, over which the sum is taken. ${{\Delta \rho }^{2}}_{\text{nuc}}$ is the nuclear contrast, $\rho$ is the scattering length density, ${N}_{0}=Z/{V}_{0}$ is the number density, ${V}_{0}$ is the unit-cell volume and ${b}_{\text{c}}$ is the coherent scattering length [24]. The scattering length density of the VC precipitate is estimated to be ${\rho }_{p}=3.475\times {10}^{14}{ \text{m}}^{-2}$ and the scattering length density of the matrix is estimated to be ${\rho }_{m}=$ $8.024\times {10}^{14}{ \text{m}}^{-2}$. Combining leads to a nuclear contrast of ${{\Delta \rho }^{2}}_{\text{nuc}}=20.69\times {10}^{28}{ \text{m}}^{-4}$.

The ferromagnetic BCC Fe matrix is magnetically ordered and its magnetic moments can be aligned in relatively low applied magnetic fields of 1 T or higher. The paramagnetic VC precipitate phase [25], which can include some limited Fe fraction, is magnetically unordered. This means that an applied magnetic field will induce a negligible alignment for the magnetic moments. Effectively, the VC precipitates therefore act as a non-magnetic phase. Randomly oriented magnetic moments will only contribute to an isotropic background. A magnetic contrast of ${{\Delta \rho }^{2}}_{\text{mag}}=24.59\times {10}^{28}{ \text{m}}^{-4}$ calculated using the following formula:

3 $${\Delta }\rho^{2}_{{{\text{mag}}}} = (\rho_{p} - \rho_{m} )^{2} = (N_{{\text{0,m}}} b_{{\text{m}}} )^{2} = (N_{{\text{0,m}}} p_{0} \mu )^{2}$$

where *b*_*m*_ is the magnetic scattering length of the ferromagnetic matrix phase, ${N}_{0,m}$ is the number density of magnetic moments in the matrix phase, *p*_0_ = 2.699 fm/*μ*_*B*_ is a constant, $\mu$ is the magnetic moment in the matrix phase in units of Bohr magneton (${\mu }_{\text{B}}$). The magnetic moment in the Fe-based matrix phase corresponds to:

4 $$\mu = \frac{{B_{{\text{s}}} }}{{N_{{\text{0,m}}} \mu_{0} \mu_{{\text{B}}} }}$$

where ${\mu }_{0}$ = 4 $\pi \times {10}^{-7}$ $\text{Tm}/\text{A}$ is the magnetic permeability in vacuum, ${\mu }_{B}=eh/4\pi {m}_{e}=9.27\times {10}^{-24}\text{J}/\text{T}$. *B*_s_ is the temperature-dependent spontaneous internal magnetic field. According to Arrott and Heinrich [26] it is given by:

5 $$B_{{\text{s}}} = \frac{{B_{0} \left( {1 - \tau } \right)^{\beta } }}{{\left( {1 - \beta \tau + A\tau^{1.5} - C\tau^{3.5} } \right)}}$$

where $\tau =T/{T}_{C}$, with *T* the temperature and *T*_*C*_ the ferromagnetic Curie temperature of the Fe-based matrix phase. The constants are *B*_0_ = 2.206 T, *T*_C_ = 1043 K, $\beta$= 0.368, *A* = 0.110, *C* = 0.129 [26]. At room temperature (*T* = 300 K) we obtain a magnetic scattering length density of ${\rho }_{m}=4.958\times {10}^{14} {\text{m}}^{-2}$. At room temperature the calculated magnetic and nuclear contrasts are comparable for VC precipitates in the Fe-based matrix with Δ*ρ*^2^_mag_/ Δ*ρ*^2^_nuc_ = 1.19.

Based on TEM and previous SANS studies [17] we assume that for interphase precipitation the shape of the VC precipitates is spherical and for random precipitation the shape of the VC precipitates corresponds to randomly oriented oblate ellipsoids. Based on the TEM images of VC precipitates (Fig. 2), where particularly the lens-shaped precipitates at the (S)GBs dominate, the aspect ratio of these oblate ellipsoids is assumed to be *η* = $${R}_{\text{p}}$$/$${R}_{\text{e}}$$ = 0.5, where $${R}_{\text{p}}$$ and $${R}_{\text{e}}$$ are the polar and equatorial radii for the ellipsoid of revolution, respectively. We then fit the $$\left(\partial\Sigma /\partial\Omega \right)\left(Q\right)$$ versus $$Q$$ SANS data using SASview [15]. The fitting was performed on both the nuclear and magnetic SANS data to obtain the volume fraction, number density and precipitate size distribution of the VC precipitates. To fit the nuclear SANS data from the samples exhibiting interphase precipitation, we use a model composed of a power law, a log-normal distribution of spherical precipitates and a constant background. In the low *Q* region (< 0.02 $${\text{\AA}}^{ - 1}$$) a power law $$K{Q}^{-n}$$ is used to fit the scattering originating from dislocations, grain boundaries and interfaces, where *K* is a pre-factor and *n* is the power [27]. At high *Q* (> 0.1 $${\text{\AA}}^{ - 1}$$$$)$$, a constant background *C* is expected that originates from incoherent scattering estimated at $${\left(\partial\Sigma /\partial\Omega \right)}_{i}=0.003 {\text{cm}}^{-1}$$ for these alloys. The scattering in the intermediate *Q* region (0.02–0.1 $${\text{\AA}}^{ - 1}$$) originates from the nanoscale VC precipitates. The SANS model for nuclear scattering with spherical precipitates corresponds to:

6 $$\left( {\frac{{\partial {\Sigma }}}{{\partial {\Omega }}}} \right)\left( Q \right) = KQ^{ - n} + \left( {\Delta \rho } \right)^{2} \mathop \smallint \limits_{0}^{\infty } D_{N} \left( r \right)V^{2} \left( r \right)P\left( {Q,r} \right){\text{dr}} + C$$

where ${\Delta \rho }^{2}$= ${({\Delta \rho }_{p} -{\Delta \rho }_{m}\text{)}}^{2}$ is the contrast, *V*(*r*) is the volume of precipitate and *r* is the radius of the precipitate. The scattering factor has been modeled as a sphere, which corresponds to *P*(*Q,r*) =|*F*(*Q,r*)|^2^ with form factor *F*(*Q,r*) = 3[sin(*Qr*)—(*Qr*)cos(*Qr*)]/(*Qr*)^3^ [28]. Polydispersity with a log-normal size distribution *D*_*N*_(*r*) = (*N*_p_/[(2π)^1/*2*^*rσ*])exp(-[ln(*r*)-ln(*r*_m_)]^2^/[2*σ*^2^]) is assumed, where *N*_p_ is the number density of precipitates, *r*_m_ is the median radius of the precipitates and *σ* is the relative width of the distribution. The data from magnetic scattering are obtained by subtracting the nuclear differential scattering cross section (*α* =  − 15° to + 15° in Eq. 1) from the total differential scattering cross section. This subtraction yields no workable data in the low *Q* region because of the absence of a magnetic contrast between the interfaces like dislocations and (S)GBs, and the Fe-matrix. Moreover, it also results in the removal of the background, which originates from incoherent scattering (0.003 ${\text{cm}}^{-1}$). Therefore, for magnetic SANS both the power law and the background terms are absent and only the precipitate scattering with the same size distribution remains, but with the magnetic instead of the nuclear contrast.

For the SANS from samples exhibiting random precipitation, the precipitates are ellipsoidal and therefore the form factor for spherical particles should be replaced by the form factor for an ellipsoid of revolution. For ellipsoids, we replace *r* in Eq. (6) by [29]:

7 $$r\left( \varphi \right) = \left[ {R_{{\text{e}}}^{2} \sin^{2} \varphi + R_{{\text{p}}}^{2} \cos^{2} \varphi } \right]^{1/2}$$

where ${R}_{\text{p}}$ and ${R}_{\text{e}}$ are the polar and equatorial radii and φ is the angle between the rotation axis of the ellipsoid of revolution and the scattering vector **Q**. The orientation of the rotation axis of the ellipsoids is assumed to be randomly distributed and the scattering factor is averaged accordingly. For the ellipsoids, an equivalent radius (assuming a sphere of equal volume) can be introduced, which corresponds to *r*_eq_ = (*R*_p_*R*_e_^2^)^1/3^. The average inter-precipitate spacing can be calculated using the number density ${N}_{p}$ obtained from performing the fitting on SANS data:

8 $$d = \left( {N_{p} } \right)^{{ - \frac{1}{3}}}$$

**Figure 3:**
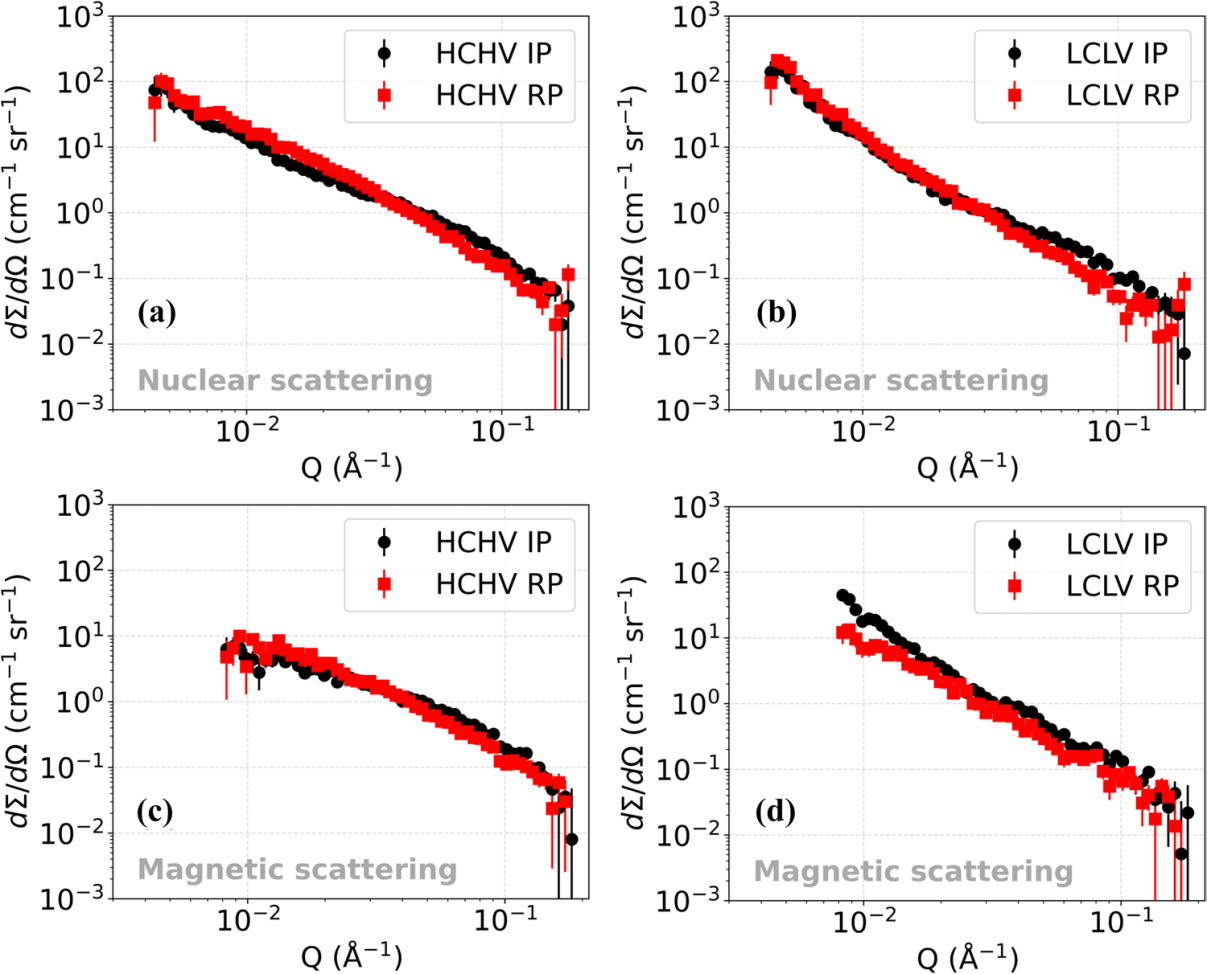
Nuclear differential scattering cross sections for the **a** HCHV and **b** LCLV alloys as a function of *Q* measured at room temperature for interphase and random precipitation during annealing at 650 °C for 20 min. The corresponding magnetic differential scattering cross sections are plotted in **c** and **d** for the HCHV and LCLV alloys, respectively.

The SANS differential scattering cross section versus *Q* plots are analyzed via model fitting in SASview and by investigating Kratky plots. In Fig. 3a, the nuclear differential scattering cross section versus *Q* is plotted for the HCHV alloy. In the high *Q* region (0.02–0.1 ${\text{\AA}}^{ - 1}$), the signal for interphase precipitation (labeled as ‘Interphase’) is stronger than that for random precipitation (labeled as ‘Random’). This implies that interphase precipitation leads to the generation of a larger quantity of smaller sized VC precipitates in comparison with random precipitation. If we focus on the low *Q* region (< 0.02 ${\text{\AA}}^{ - 1}$), we see a stronger signal for random precipitation. This could mean a greater amount of large scatterers like large precipitates, grain boundaries and dislocations.

In Fig. 3b the nuclear SANS for the LCLV alloy is shown. In the high *Q* region, we see a stronger signal for interphase precipitation compared to random precipitation, in agreement with the results for the HCHV alloy. In the low *Q* region, we see an identical signal for both random and interphase precipitation, indicating the presence of a similar amount of large precipitates, grain boundaries and dislocations.

From the magnetic differential scattering cross section versus *Q* data for the HCHV alloy in Fig. 3c, we can make the following observations. In the high *Q* region, we see a stronger signal for interphase precipitation compared to random precipitation. The curves look comparable to the ones for the nuclear scattering from the HCHV alloy in Fig. 3a. Similarly, for the magnetic differential scattering cross section versus *Q* data from the LCLV alloy in Fig. 3d, we see a stronger signal from interphase precipitation compared to random precipitation, indicating that more VC precipitates are formed during interphase precipitation.

The small-angle scattering in the low *Q* region (< 0.02 ${\text{\AA}}^{ - 1}$) of the studied alloys can be described by a power law $\left(\partial\Sigma /\partial\Omega \right)\propto K{Q}^{-n}$ where *n* is ranging from 2 to 4. It has been found that the strain fields associated with dislocations give rise to the scattering in the low *Q* region [27, 30]. In previous experiments performed to study the small-angle scattering from dislocation structures in deformed metals, it was found that bulk averaged scattering from edge dislocations gives rise to a ${Q}^{-3}$ power law dependence [31–33]. Long and Levine [27] performed Ultra Small-Angle X-ray Scattering (USAXS) experiments to study the scattering from dislocations in metals. Their experimental results were in agreement with theory, which predicts that scattering from an individual dislocation shows ${Q}^{-2}$ dependence, whereas scattering from dislocation dipoles shows ${Q}^{-3}$ dependence and scattering from sharp dislocation walls shows ${Q}^{-4}$ dependence. The scattering from sharp GB and SGB shows the same scattering behavior as found for the scattering from sharp dislocation walls. For smeared dislocation walls, GB and SGB, the value of *n* decreases and becomes less than 4.

**Figure 4:**
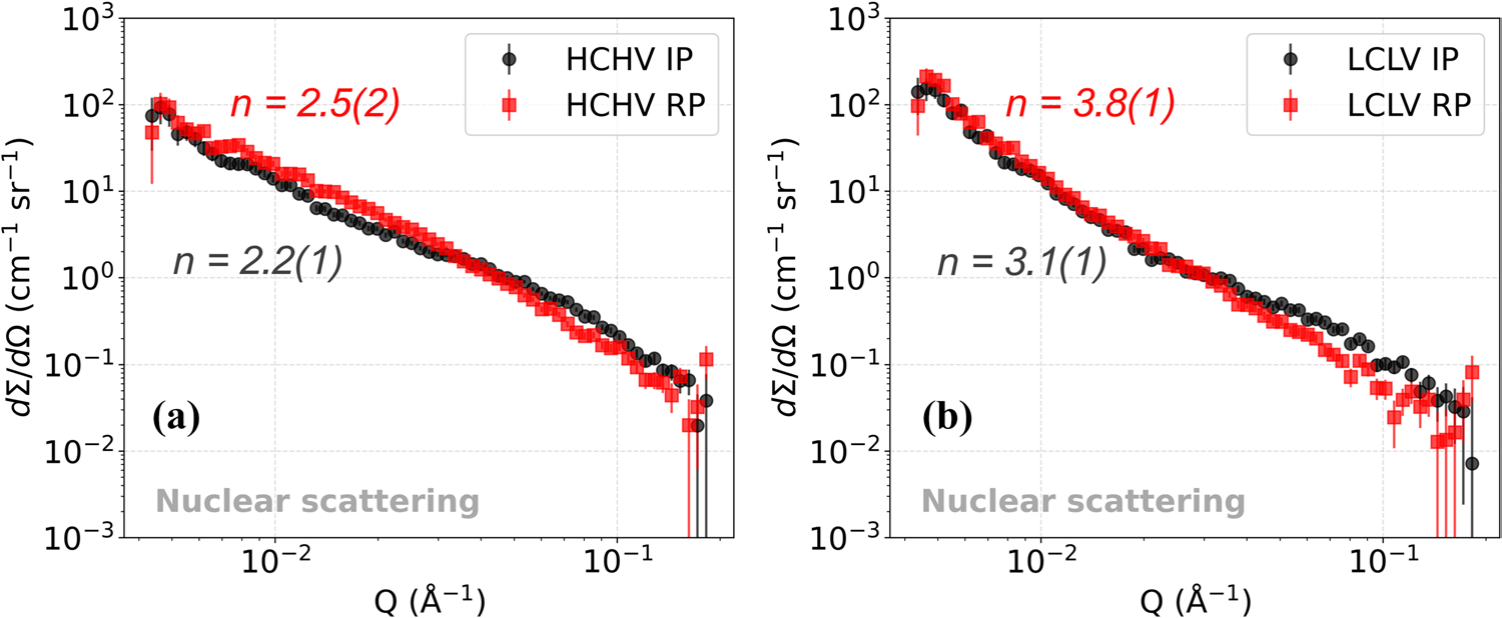
Nuclear differential scattering cross sections from the HCHV and LCLV alloys with interphase precipitation (IP) and random precipitation (RP), where the low-Q scattering power law is indicated.

As illustrated in Fig. 4a, it was found for the HCHV alloy that the nuclear SANS curve in the low *Q* region shows ${Q}^{-2.2}$ and ${Q}^{-2.5}$ dependence for IP and RP, respectively, whereas in case of the LCLV alloy a ${Q}^{-3.1}$ and ${Q}^{-3.8}$ dependence was found for IP and RP, respectively (see Fig. 4b). The value of the exponent *n* comes out to be smaller than 4 in both the HCHV and LCLV alloys. A possible reason for this could be that the contribution to the SANS signal from VC precipitates is dominant over that from the interfaces (dislocations, GBs and SGBs). This complicates the task of interpreting parameters like the exponent *n* and pre-factor *K* obtained by fitting our SANS model (Eq. 6) to the data. The degree of this dominance of SANS from VC precipitates is higher in HCHV as compared to that in LCLV. The reason for this is the higher number of VC precipitates present in HCHV. Whereas in LCLV, a lower number of VC precipitates results in a weaker dominance of the signal from VC precipitates over that from the interfaces. This is reflected in the extracted value of n (3.1(1) and 3.8(1)), which is higher in LCLV than that obtained in HCHV. This possibly indicates scattering from GBs/SGBs in LCLV. Moreover, it is known from previous studies that carbon tends to segregate at the interfaces like dislocations and GBs/SGBs [34], which would make the formation of VC precipitates easier on these sites. This could possibly make the interfaces rough which could also cause a deviation from the expected ${Q}^{-n}$ behavior of the SANS signal. Unfortunately, the $Q$ range which corresponds to scattering from dislocations, GB and SGB is limited and is influenced by the signal from VC precipitates as well. This makes the task of fitting and interpretation complicated.

**Figure 5:**
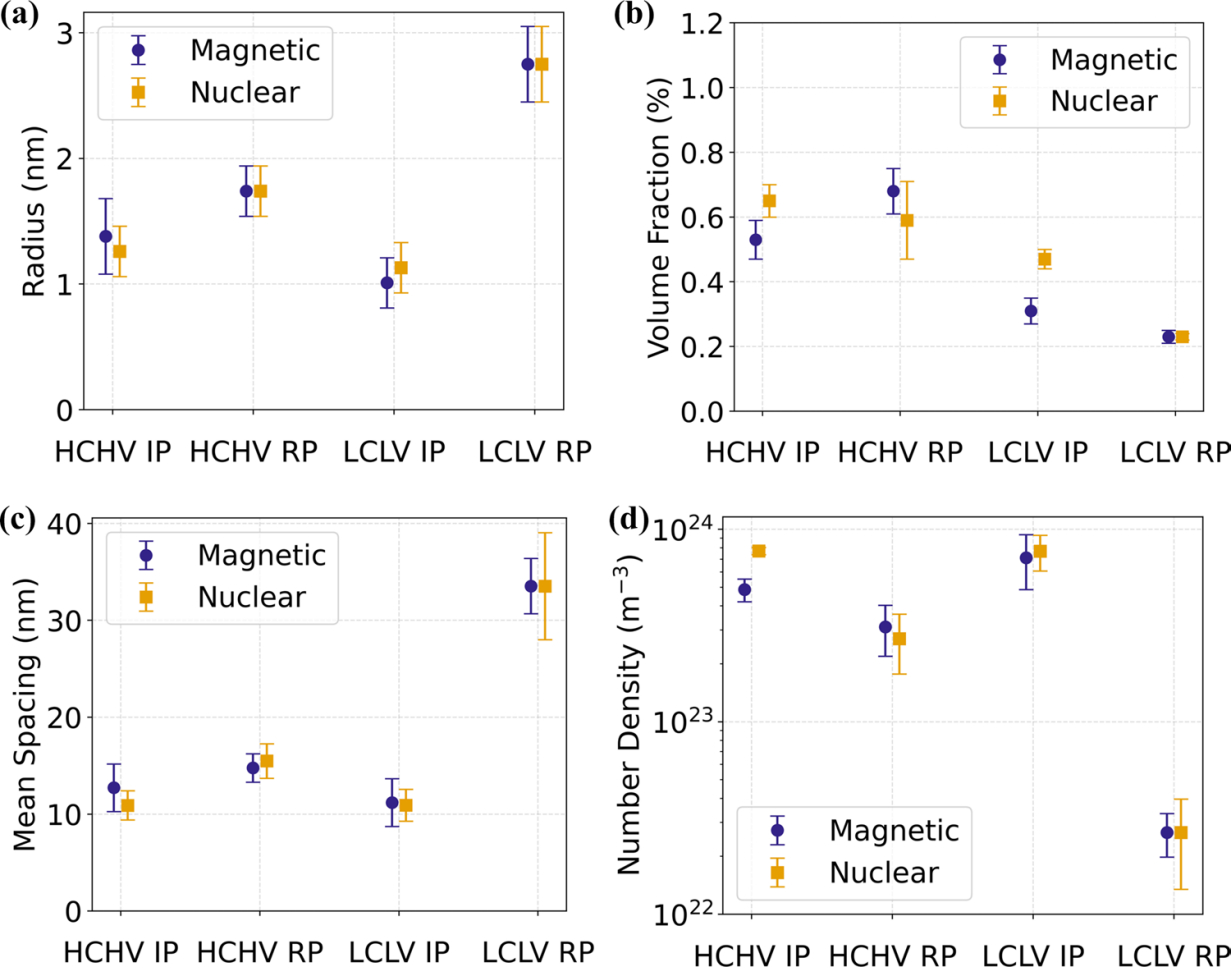
Calculated VC precipitate **a** equivalent radius, **b** volume fraction, **c** inter-precipitate spacing and **d** number density, obtained by a model fit of the nuclear and magnetic differential scattering cross sections versus *Q*.

The model fitting of the nuclear and magnetic SANS reveals noteworthy differences in the equivalent radius of the precipitates across the studied steel samples as depicted in Fig. 5. These results shed light on the effectiveness of different precipitation mechanisms in influencing the size distribution. The samples with interphase precipitation display smaller precipitate radii compared to the samples with random precipitation. A possible reason for this difference in the studied samples could be the high density of the activated nucleation sites for interphase precipitation in comparison with random precipitation. This probably leads to the formation of a higher number of smaller sized precipitates for interphase precipitation. Interphase precipitation involves precipitate formation during the austenite-to-ferrite phase transformation, with precipitates being nucleated on the migrating interphase between austenite and ferrite [18] which is accompanied by the diffusion of V and C atoms. As all the austenite phase will be transformed to the ferrite phase, the migrating austenite-ferrite interface potentially travels through the complete microstructure. As a result, the a large sample volume is available for nucleation of precipitates at the moving austenite/ferrite interphase in IP. For random precipitation, fewer activated nucleation sites coupled with the formation of cementite [35], could result in the consumption of carbon atoms which might reduce the driving force for VC formation. Moreover, at the interfaces (dislocations and (S)GBs), which are plenty in case of the RP samples, because of faster precipitation kinetics larger precipitates could form as is evident in Fig. 5a. This can lead to the formation of larger but fewer VC precipitates in the RP samples, which can also be observed by looking at the inter-precipitate spacing and the precipitate number density in Figs. 5c and d, respectively.

If we focus on the volume fraction of VC precipitates in Fig. 5b obtained from fitting the nuclear and magnetic $\left(\partial\Sigma /\partial\Omega \right)\left(Q\right)$ versus *Q* data, we can observe the effects of different precipitation mechanisms and variations in the carbon and vanadium concentrations on the volume fraction. By increasing the carbon and vanadium concentrations, higher VC volume fractions are obtained, indicating a direct correlation between the carbon and vanadium concentrations and the VC volume fraction. Moreover, the volume fractions of VC in all the samples are below the calculated equilibrium values of *f*_eq_ = 1.08% and *f*_eq_ = 0.56% at 650 °C for the HCHV and LCLV alloys, respectively.

**Figure 6:**
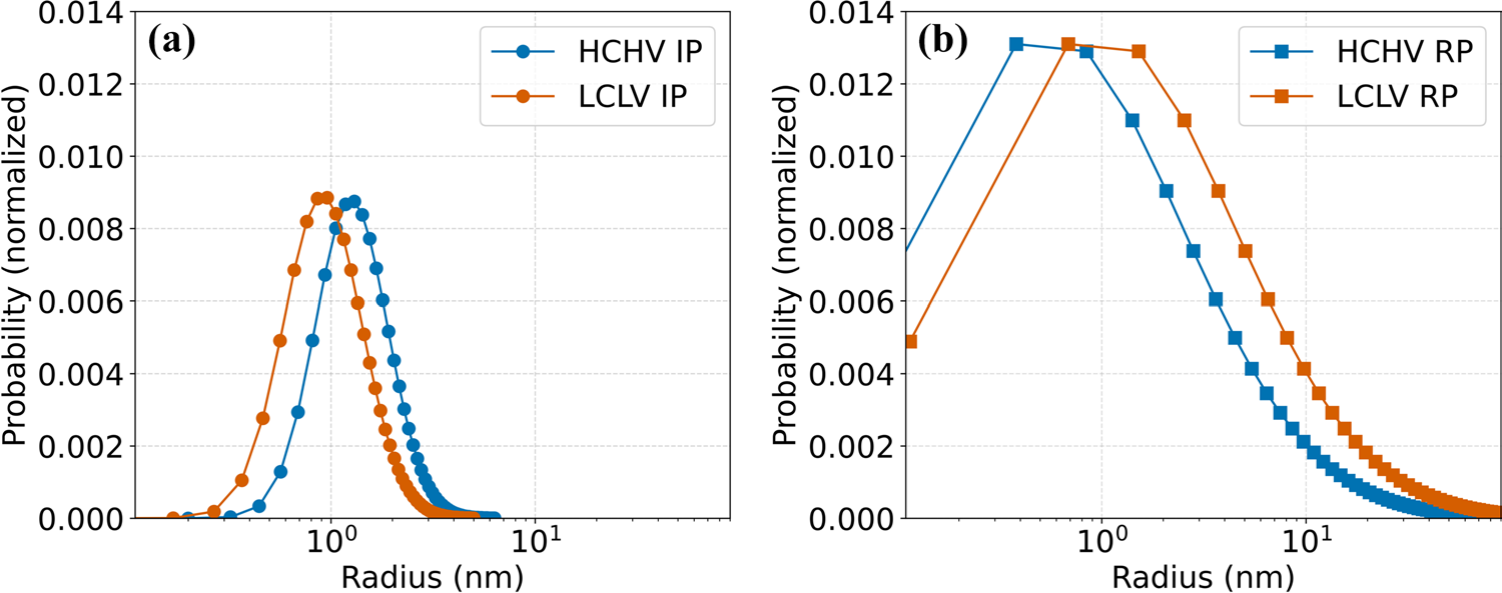
Normalized size distribution for **a** the radius of spherical VC precipitates in interphase precipitation (IP) and **b** the equivalent radius of ellipsoidal VC precipitates in random precipitation (RP).

It is possible to estimate the size distribution of VC precipitates in the studied alloys. For this purpose, we use the following equation to calculate the assumed log-normal size distribution:

9 $$f\left( r \right) = \frac{1}{Nr\sigma }{\text{exp}}\left( { - \frac{1}{2}\left( {\frac{{\ln \left( r \right) - {\text{ln}}\left( {r_{{\text{m}}} } \right)}}{\sigma }} \right)^{2} } \right)$$

where $f\left(r\right)$ is the probability density function (PDF) of the log-normal distribution, $N$ is a normalization factor, $r$ is the (equivalent) radius (equal to the radius for spheres), ${r}_{\text{m}}$ is the median value of (equatorial) radius, $\sigma$ is the relative width of the log-normal distribution. In Fig. 6, we can see the derived log-normal size distribution of VC precipitates. A broad size distribution of VC precipitates can be observed in the RP samples compared to the IP samples. Furthermore, we observe that the LCLV RP sample exhibits a size distribution at larger radius values compared to the HCHV RP sample, whereas the opposite is the case with IP samples. These observations are consistent with TEM analysis, as depicted in Fig. 2. Specifically, in Fig. 2c, d, g and h, we can observe the presence of both large and small VC precipitates in the CERs, where the large VC precipitates at GBs/SGBs are more prevalent in the RP samples in comparison to the IP samples.

**Figure 7:**
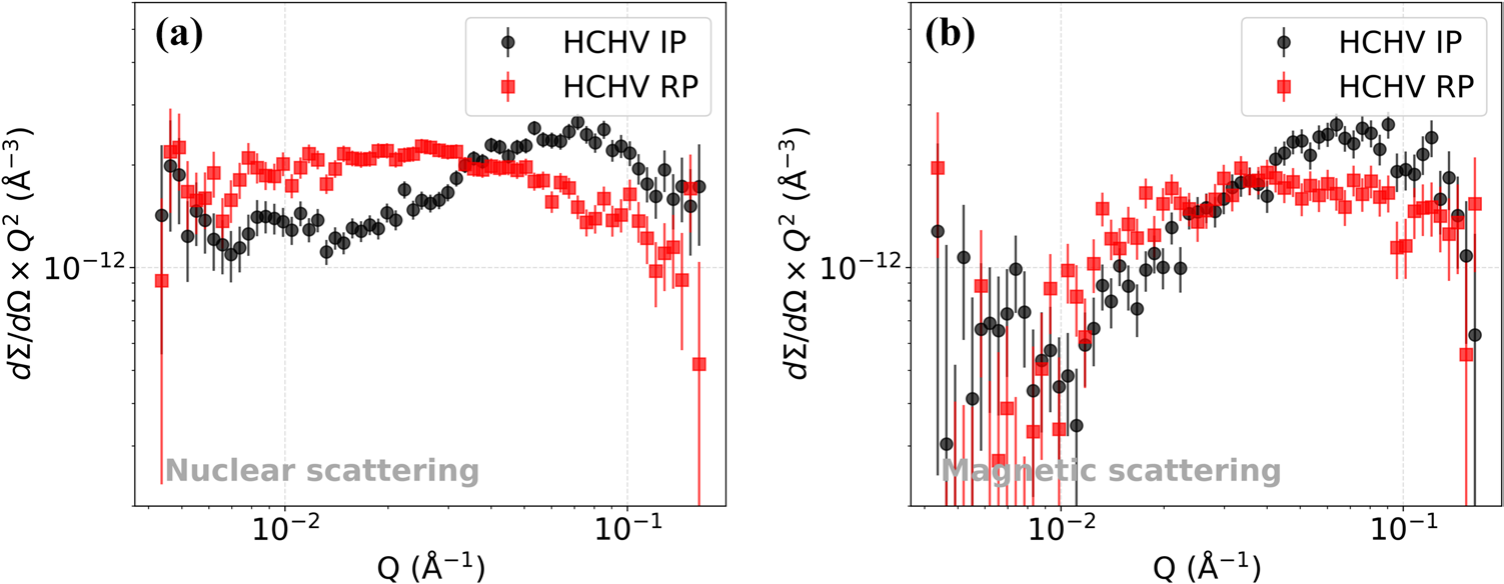
Kratky plots indicating **a** the nuclear and **b** magnetic SANS data of $\left(\partial\Sigma /\partial\Omega \right){Q}^{2}$ versus *Q* for the HCHV alloy with interphase precipitation and random precipitation.

It is also possible to analyze the precipitate volume fraction ${f}_{v}$ using Kratky plots. This method possesses the benefit that the determination of the volume fraction is independent of the shape of the precipitates. In the Kratky plots shown in Fig. 7 the differential scattering cross section $\left(\partial\Sigma /\partial\Omega \right)$ is multiplied with ${Q}^{2}$ and plotted vs *Q*. Integration of the area under the Kratky plot results in the invariant ${Q}_{0}$ [21]:

10 $$Q_{0} = \mathop \smallint \limits_{0}^{\infty } \left( {\frac{d\Sigma }{{d\Omega }}} \right)Q^{2} dQ = 2\pi^{2} \Delta \rho^{2} f_{v} \left( {1 - f_{v} } \right)$$

where ${\Delta \rho }^{2}$ is the SANS contrast and ${f}_{v}$ is the volume fraction of the VC precipitates. For dilute systems ${f}_{v}\left(1-{f}_{v}\right)\approx {f}_{v}$.

We can observe the differences between interphase and random precipitation by analyzing the Kratky plots shown in Fig. 7a and b, respectively. These plots are generated by using the nuclear and magnetic SANS from the HCHV alloy. We observe a difference in the peak value, with the peak occurring at lower *Q* values in the case of random precipitation compared to interphase precipitation. This suggests a variation in size distribution for the VC precipitates formed through different precipitation mechanisms. A peak at lower *Q* value implies a larger average precipitate size, whereas a peak at higher *Q* value indicates a smaller average precipitate size. This analysis is consistent with the radius values derived from fitting the $(\partial\Sigma /\partial\Omega )$ versus *Q* data using SASview and the TEM results, as depicted in Figs. 5a and 2, respectively.

**Figure 8:**
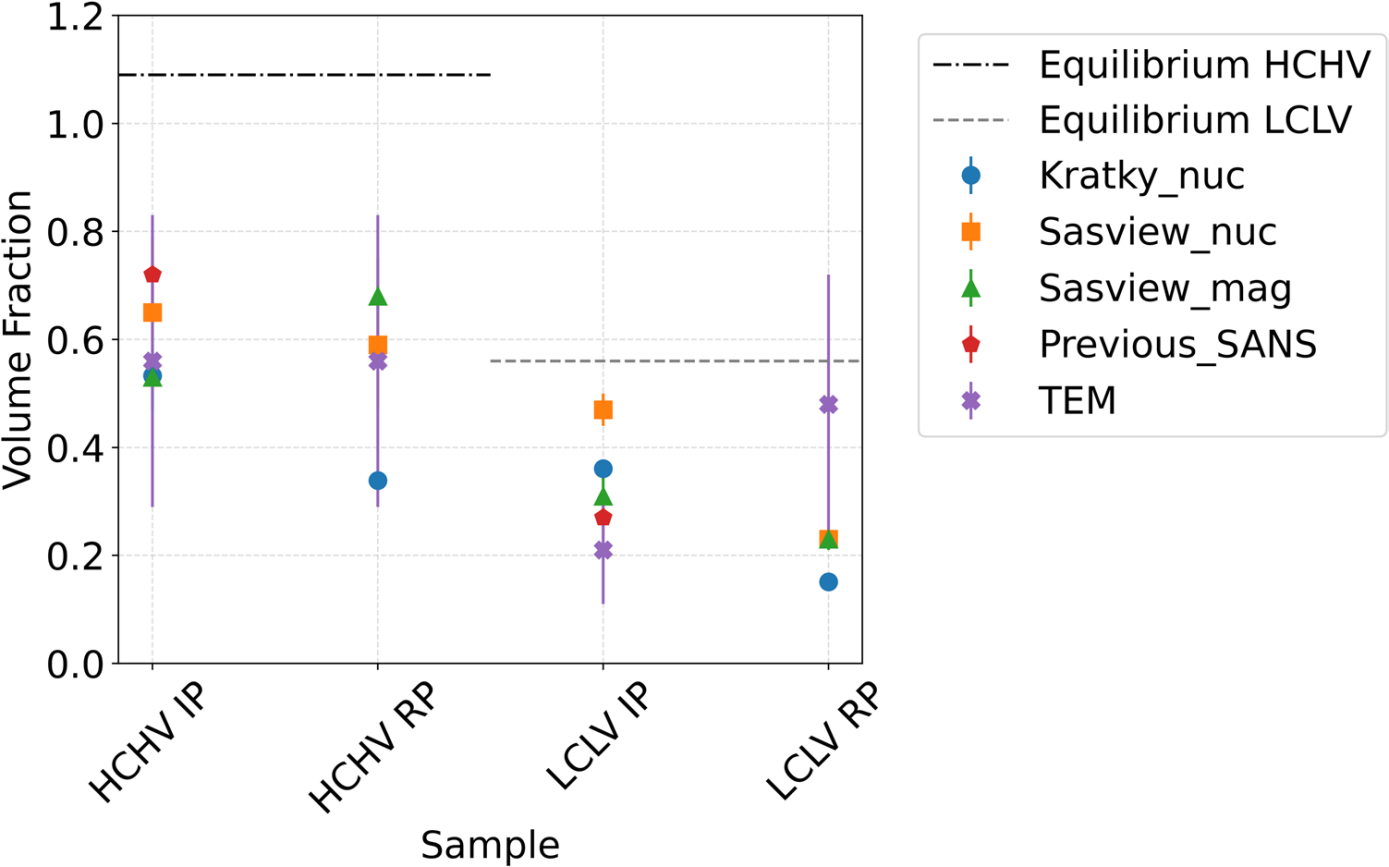
Comparison of the VC precipitate volume fraction values obtained by Kratky analysis, model fitting (SASview) on nuclear and magnetic SANS, previous ex situ SANS experiments [17], TEM analysis and the equilibrium value using ThermoCalc.

Integrating the area under the Kratky plots gives the volume fraction of VC precipitates, which comes out to be higher in case of interphase precipitation for both HCHV and LCLV alloys. Furthermore, a higher value of volume fraction is obtained in the samples with higher carbon and vanadium contents (HCHV). In Fig. 8, these values are compared with the values obtained by using the SANS model fit of the nuclear and magnetic $(\partial\Sigma /\partial\Omega )$ versus Q curves, previous ex situ SANS studies [17] and TEM analysis.

When comparing the methods to characterize the VC precipitate volume fraction, it is appropriate to consider their respective advantages and limitations. The major benefit of using TEM is that we get input regarding the shape of the VC precipitates which is important for interpreting the SANS data. The input from TEM is then used to construct a model (Eq. 6) which accurately describes the neutron beam that is scattered from the nano-VC precipitates. By fitting this model (Eq. 6) to the experimental SANS data, we extract the VC precipitate volume fraction. TEM, therefore, acts as a complementary technique to SANS. There are several drawbacks of only using TEM to study VC precipitation in nanosteels. For instance, the TEM analysis is confined to a localized region of the sample and therefore the observations may potentially deviate from the material’s bulk behavior. If SANS is used without the input regarding the precipitate morphology from the TEM, then we can resort to the Kratky method for data analysis which is a shape independent data analysis method. This method, however, has its limitations when it comes to an accurate quantification of the VC precipitate volume fraction and average size. This limitation arises because of the uncertainty involved in selecting the *Q* range in which the integration of the Kratky curves is performed. When it comes to model fitting, then this is the best choice to perform an accurate SANS data analysis and extract quantitative parameters such as the volume fraction and the size distribution. It should be mentioned that it can be challenging to deconvolute the contribution from the dislocations, (S)GBs and the VC precipitates to the SANS signals.

Figure 8 compares the VC precipitate volume fraction as obtained from different SANS data analysis methods, TEM, literature and theoretical equilibrium calculations. The volume fraction values obtained from Kratky method seems to be underestimated, when compared to those obtained from model fitting and those obtained by Ioannidou C. et al. [17, 18], except for LCLV IP. This could easily be because of an under- or over-estimation of the *Q* range that was used to calculate the invariant, which was then used to calculate the VC precipitate volume fraction*.* The VC precipitate volume fraction values obtained from model fitting are obtained by taking into consideration the SANS from dislocations and (S)GBs as well. It is possible to deconvolute the SANS from VC precipitates and SANS from dislocations and (S)GBs by adding the two terms that describe their SANS in the model. This results in the volume fraction values that are higher than those obtained from Kratky analysis, but are comparable to the values found in literature (red pentagons in Fig. 8). The volume fraction values obtained from theoretical equilibrium calculations (see Table 2) are higher than those obtained in this study because the studied samples were annealed for a period of 20 min which is far from equilibrium. Moreover, the theoretical equilibrium calculations predict the presence of Fe atoms in the VC precipitates as well (more on this in the discussion section). This is not taken into consideration while calculating the neutron contrast. In this study, the precipitate volume fractions were also calculated using the TEM as well, and a reasonable agreement between the TEM and SANS data was observed.

**Table 2:** Equilibrium composition of the precipitates

Steel		C	Fe	V	Mn
HCHV	wt.% at.%	16.84 46.55	12.58 7.48	70.16 45.72	0.42 0.25
LCLV	wt.% at.%	16.84 46.58	14.04 8.35	68.62 44.76	0.51 0.31

## Discussion

It is found that for 20 min annealing at 650 °C a higher number density of nanoscale VC precipitates is formed during interphase precipitation compared to random precipitation (Fig. 5c). This implies that more nucleation sites are active during the interphase precipitation. The higher number density, and consequently a smaller inter-precipitate spacing, are in line with a smaller size of the VC precipitates when the precipitate volume fractions are comparable. This implies that in case of the studied random precipitation samples, we expect the pinning distance to be greater and hence the strengthening to be weaker. With the obtained precipitate size distribution, it becomes possible to estimate the precipitation strengthening originating from interphase precipitation and from random precipitation. For this we use the Ashby-Orowan equation [36], which relates the increase in strength to the volume fraction and radius of the precipitates:

11 $$\Delta \sigma = \frac{{0.538Gb\sqrt {f_{v} } }}{2r}\ln \frac{r}{b}$$

where $\Delta \sigma$ is the increase in strength resulting from precipitation hardening, *G* is the shear modulus of the matrix, *b* is the Burgers vector, ${f}_{v}$ is the volume fraction of the precipitates, and *r* is the average (equivalent) radius of the precipitates.

**Figure 9:**
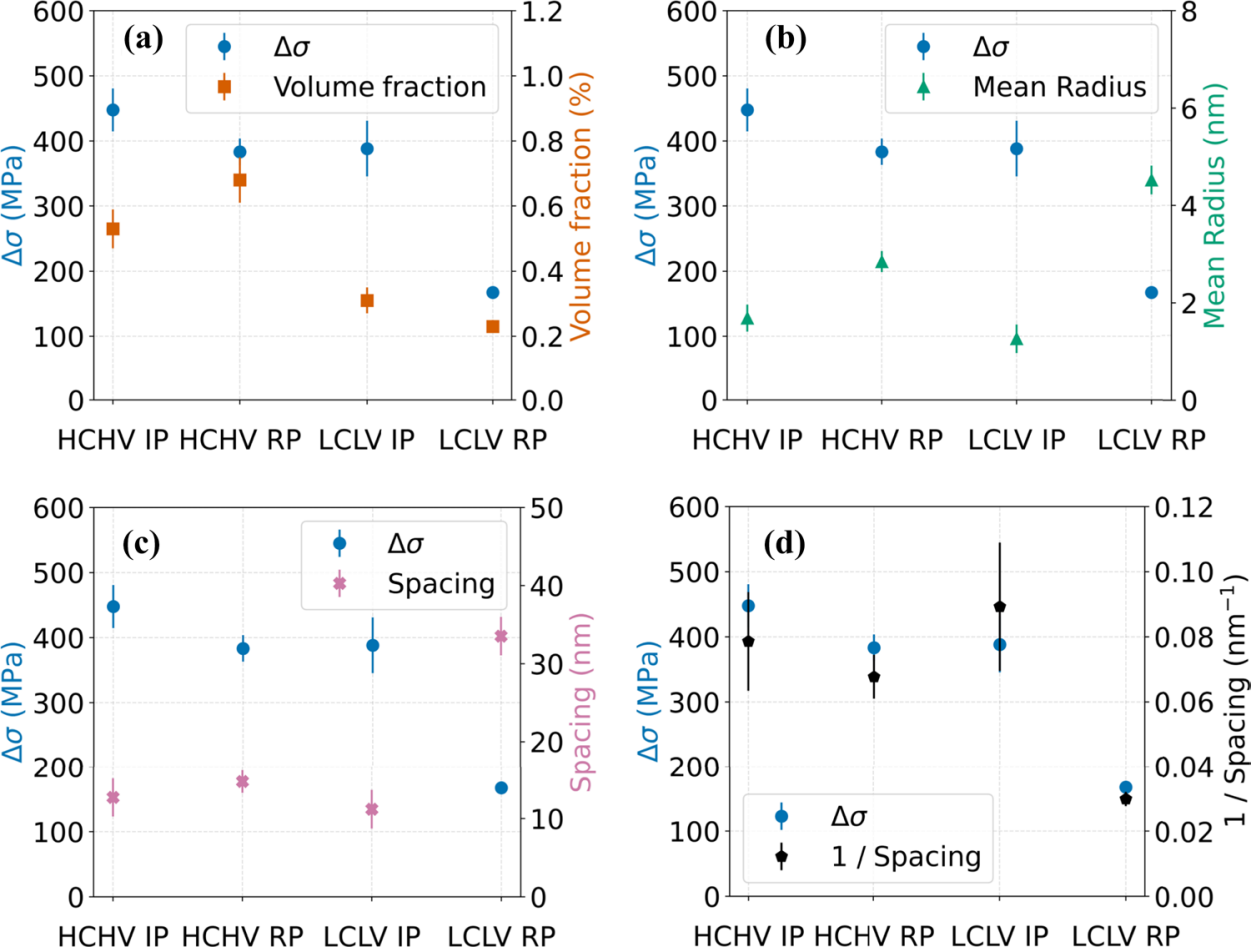
Estimated increase in strength by VC precipitate formation in the HCHV and LCLV steels under interphase precipitation (IP) and random precipitation (RP) conditions, based on the analysis from the magnetic SANS contribution, plotted against **a** VC volume fraction, **b** mean radius, **c** inter-precipitate spacing and **d** inverse inter-precipitate spacing.

For *G* = 81600 MPa and $b=a\sqrt{3}/2$, with $a = 2.86 {\text{\AA}}$ being the lattice parameter of the BCC Fe-based matrix, it becomes possible to calculate the increase in strength by VC precipitate formation. As shown in Fig. 9a, the increase in strength in the samples exhibiting interphase precipitation is found to be larger than that of the samples exhibiting random precipitation.

For the samples with interphase precipitation (IP), the increase in strength by precipitation is identical for the HCHV and LCLV alloys (Fig. 9a), indicating a weak dependence on the carbon and vanadium concentrations. This is because the increase in strength mainly depends on the VC precipitate inter-precipitate spacing, which is comparable in both HCHV-IP and LCLV-IP steels (Fig. 5d). According to Ashbey-Orowan model for precipitation strengthening (Eq. 11), we see that the strength increase is proportional to $\sqrt{{f}_{v}}/r$. On calculating this ratio for HCHV IP and LCLV IP, we obtain similar values which are 0.053 and 0.055 ${\text{nm}}^{-1}$, respectively. The increase in precipitate volume fraction by the addition of carbon and vanadium is compensated by an increase in the precipitate size, resulting in a comparable strengthening effect. The correlation between the strength increase, the precipitate volume fraction, the mean precipitate radius, the inter-precipitate spacing and inverse inter-precipitate spacing is shown in Fig. 9a–d, respectively, whereas, for the samples with random precipitation (RP), the increase in carbon and vanadium concentrations results in an increase in VC precipitation strengthening by a factor of $2$, indicating almost linear dependence of precipitation strengthening on the carbon and vanadium concentrations.

The experimental volume fractions ${f}_{v}$ obtained by SANS are, as expected, consistently lower than the equilibrium values predicted by Thermo-Calc, indicating that annealing for 20 min at 650 °C is not sufficient to reach the equilibrium precipitate fraction in the studied alloys. This means that at the end of the annealing step there is still vanadium and carbon dissolved in the matrix. The precipitate volume fractions for the samples with interphase precipitation seem closer to the corresponding equilibrium values compared to those of the samples with random precipitation, suggesting a higher precipitation efficiency in the annealing step (see Fig. 8).

A high number density of VC precipitates for interphase precipitation in comparison to random precipitation can be observed in Fig. 5d. A possible reason for the relatively low density of nucleation sites for the random precipitation samples could be related, in part, to the formation of a mixed bainitic/martensitic microstructure in these samples during cooling at the rate of 15 °C/s after austenitization (1100 °C for HCHV and 1050 °C for LCLV). Compared to a fully martensitic microstructure at the start of the precipitation process, a mixed bainitic/martensitic microstructure may yield a lower density of dislocations/interfaces that could act as potential nucleation sites for VC precipitation during the annealing step at 650 °C [37]. Moreover, the re-heating rate applied after cooling the samples to room temperature influences the available dislocations/interfaces present at the start of the isothermal precipitation at 650 °C. A faster re-heating is expected to result in a more effective preservation of the dislocations/interfaces initially present at room temperature [38]. Another possible reason for a lower number density of VC precipitates in RP samples could be the formation of cementite [35], which consumes some of the carbon and therefore reduces the driving force for VC precipitate formation.

Generally, three different VC precipitate shapes are observed in the studied samples, lens-shaped precipitates at GBs/SGBs, prolate ellipsoidal ones at dislocations, and spherical precipitates within the matrix (Fig. 2). The VC precipitates reach a relatively coarse ellipsoidal or lens shape possibly because of pipe diffusion along dislocations or grain-boundary diffusion along (sub-)grain boundaries of the matrix, respectively [10]. Further systematic studies are required to better understand the formation mechanisms of these differently shaped VC nano-precipitates.

An analysis of the *A*-factor, which refers to the relative strength of the magnetic and the nuclear scattering from the VC precipitates, should indicate if the assumed 1:1 stoichiometry of the VC precipitates is experimentally confirmed by the SANS results. Using the experimental SANS data, it becomes possible to calculate the *A-*factor to get an idea about any possible compositional changes in the VC precipitates when the studied samples are annealed for 20 min at 650 °C. The formula used to calculate the *A*-factor is:

12 $$A = \frac{{\left( {\frac{{\partial {\Sigma }}}{{\partial {\Omega }}}} \right)_{{{\text{nuc}}}} \left( Q \right)}}{{\left( {\frac{{\partial {\Sigma }}}{{\partial {\Omega }}}} \right)_{{{\text{mag}}}} \left( Q \right)}} = \frac{{{\Delta }\rho^{2}_{{{\text{nuc}}}} }}{{{\Delta }\rho^{2}_{{{\text{mag}}}} }}$$

**Figure 10:**
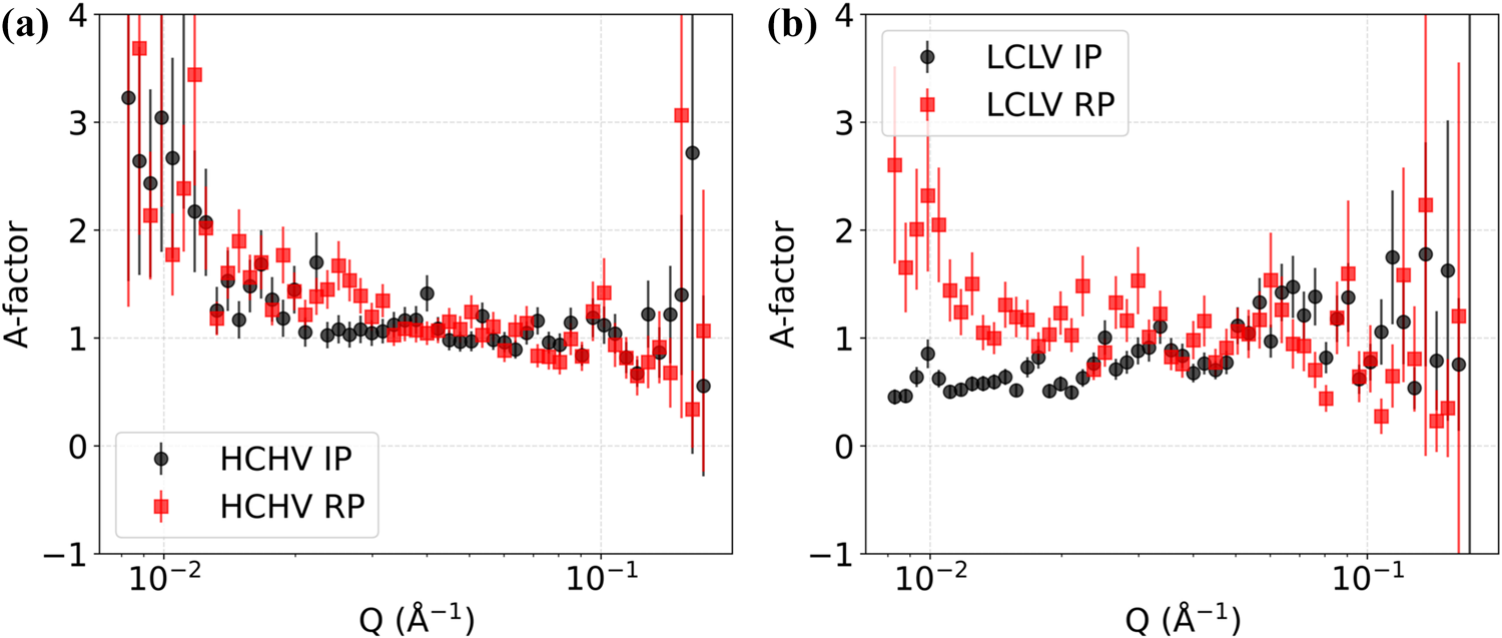
*A*-factor profiles for **a** HCHV alloy and **b** LCLV alloy.

**Figure 11:**
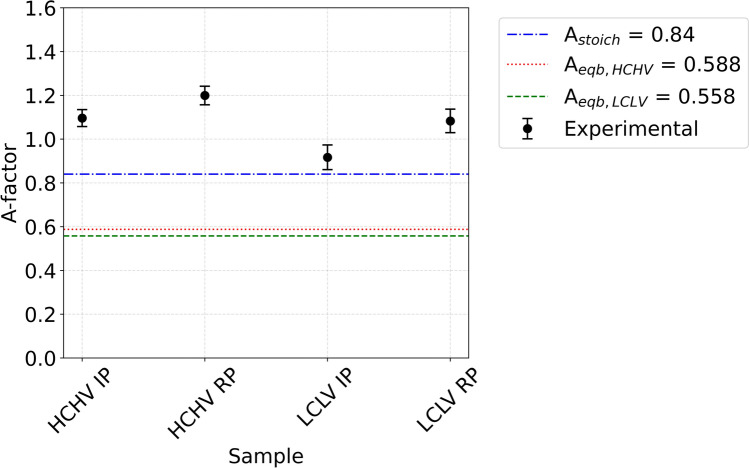
Comparative plot showing A-factor experimental (weighted mean value of the A-factors), *A*_stoichiometric_, *A*_eqb,HCHV_ and *A*_eqb,LCLV_.

${{\Delta \rho }^{2}}_{\text{nuc}}$ and ${{\Delta \rho }^{2}}_{\text{mag}}$ are calculated assuming the precipitates contain only V and C atoms. The presence of Fe atoms in the precipitate would decrease${{\Delta \rho }^{2}}_{\text{nuc}}$, while ${{\Delta \rho }^{2}}_{\text{mag}}$ would remain the same, eventually decreasing the A-factor. When stoichiometric VC is assumed, the value of the calculated *A*-factor is *A*_stoichiometric_ = 0.84. In Fig. 10 we can see the plotted A-factor values for HCHV and LCLV. The weighted mean of experimental A-factor values ($\overline{A }$) for HCHV IP, RP and LCLV IP, RP are 1.10 ± 0.04, 1.20 ± 0.04 and 0.92 ± 0.06, 1.08 ± 0.05, respectively (see Fig. 11). These values are calculated in the *Q* range from *Q* = 0.002–0.007, to avoid data points with large error bars. The mean of experimental A-factor values are relatively higher in the HCHV alloy as compared to those in the LCLV alloy, indicating a possible variation in precipitate composition, as was previously observed by Ioannidou et al. [39]. The A-factor values seem to be higher in the samples with RP as compared to those with IP, indicating a possible sensitivity for the precipitation mechanism. The weighted mean ($\overline{A }$) of the *A*-factor turns out to be higher than the *A*_eqb,HCHV_, *A*_eqb,LCLV_ and *A*_stoichiometric_ estimates for both alloys. Based on the $\overline{A }$ values it can be concluded that the composition of the VC precipitates deviates in all samples from the equilibrium composition predicted by ThermoCalc (see Table 2), which predicts 12.58 and 14.04 wt% Fe in the precipitates formed in HCHV and LCLV, respectively. This means that annealing at 650 °C for 20 min results in a limited incorporation of Fe atoms in the VC precipitates.

## Conclusions

In this study, we conducted SANS experiments on two nanosteel alloys with different carbon and vanadium concentrations subjected to two different heat treatments, leading to either interphase precipitation or random precipitation of nanoscale VC precipitates. The conclusions can be drawn as follows:

1. For interphase precipitation, TEM observations mainly reveal spherical-shaped VC precipitates. In contrast, VC precipitates resulting from random precipitation exhibit a lens shape, characterized by an oblate ellipsoid of revolution, with an average aspect ratio of 0.5. We found that VC precipitates formed through interphase precipitation are smaller in size and demonstrate a higher number density as compared to those formed through random precipitation. Additionally, higher carbon and vanadium contents result in a higher volume fraction of VC nano-precipitates.
2. In comparison to random precipitation, interphase precipitation results in a higher number of activated nucleation sites, as evidenced by higher number density values and smaller VC precipitate sizes. A possible reason for the lower number density of VC precipitates in the case of random precipitation may be attributed, in part, to the applied slow cooling and reheating rates (both 15 °C/s), which weakens the creation and preservation of dislocations within the samples. Another possible reason for the lower VC number density via random precipitation could be a limited formation of cementite, which would consume carbon and would therefore reduce the driving force for VC precipitation.
3. Adding carbon and vanadium does not seem to increase VC precipitation strengthening when the mechanism of precipitation is interphase, whereas when the mechanism of precipitation is random, then adding carbon and vanadium seems to increase the VC precipitation strengthening, indicating a direct correlation between carbon and vanadium concentration and precipitation strengthening. This highlights the significance of precipitation mechanism in determining the mechanical properties of nanosteels.
4. The *A*-factor analysis shows a possible dependence of the VC precipitate composition on the vanadium and carbon concentration. A higher $\overline{A }$ value is obtained in the HCHV alloy samples in comparison with the LCLV alloy samples. The VC precipitate composition seems to be sensitive to the precipitation mechanism for a annealing time of 20 min at 650 °C.

Overall, our study provides valuable insights into the effects of different heat treatments and associated precipitation mechanisms on the size distribution, volume fraction, number density and composition of the nanoscale VC precipitates in nanosteels, thereby contributing to a deeper understanding of their formation mechanism and strengthening effects. One key finding in this study is related to the effect of carbon and vanadium addition on the precipitate number density when the precipitation mechanism is interphase (IP). From our SANS results, we observed that the precipitate number density in the LCLV IP sample is comparable to or higher than that in the HCHV IP sample, meaning that the addition of carbon and vanadium does not necessarily increase the precipitate number density. The number density also seems to depend on the mechanism of precipitation. In this study, we see a considerable effect of carbon and vanadium addition on the number density when the mechanism is RP, but that is not the case when it comes to IP. This information can be useful for designing nanosteels, particularly when it comes to saving natural resources like vanadium, reducing carbon footprint by using a lower amount of carbon and at the same time not compromising on the level of yield strength.

## Data Availability

The data are available from the corresponding author upon reasonable request.
